# Clinical, pathologic, and genomic characteristics of two pediatric glioneuronal tumors with a *CLIP2::MET* fusion

**DOI:** 10.1186/s40478-024-01776-1

**Published:** 2024-04-22

**Authors:** Nicholas Chapman, Joshua Greenwald, Jolee Suddock, Dong Xu, Alexander Markowitz, Maeve Humphrey, Jennifer A Cotter, Mark D Krieger, Debra Hawes, Jianling Ji

**Affiliations:** 1 Division of Neurosurgery, Neurological Institute, Children’s Hospital Los Angeles, Los Angeles, CA USA; 2 Department of Pathology and Laboratory Medicine, Children’s Hospital Los Angeles, Los Angeles, CA USA; 3 Keck School of Medicine, University of Southern California, Los Angeles, CA USA

## Abstract

Integration of molecular data with histologic, radiologic, and clinical features is imperative for accurate diagnosis of pediatric central nervous system (CNS) tumors. Whole transcriptome RNA sequencing (RNAseq), a genome-wide and non-targeted approach, allows for the detection of novel or rare oncogenic fusion events that contribute to the tumorigenesis of a substantial portion of pediatric low- and high-grade glial and glioneuronal tumors. We present two cases of pediatric glioneuronal tumors occurring in the occipital region with a *CLIP2*::*MET* fusion detected by RNAseq. Chromosomal microarray studies revealed copy number alterations involving chromosomes 1, 7, and 22 in both tumors, with Case 2 having an interstitial deletion breakpoint in the *CLIP2* gene. By methylation profiling, neither tumor had a match result, but both clustered with the low-grade glial/glioneuronal tumors in the UMAP. Histologically, in both instances, our cases displayed characteristics of a low-grade tumor, notably the absence of mitotic activity, low Ki-67 labeling index and the lack of necrosis and microvascular proliferation. Glial and neuronal markers were positive for both tumors. Clinically, both patients achieved clinical stability post-tumor resection and remain under regular surveillance imaging without adjuvant therapy at the last follow-up, 6 months and 3 years, respectively. This is the first case report demonstrating the presence of a *CLIP2*::*MET* fusion in two pediatric low-grade glioneuronal tumors (GNT). Conservative clinical management may be considered for patients with GNT and *CLIP2*:*MET* fusion in the context of histologically low-grade features.

**Supplementary Information:**

The online version contains supplementary material available at 10.1186/s40478-024-01776-1.

## Introduction

Emerging technological advancements have played a crucial role in delineating a classification system for tumors affecting the central nervous system (CNS). The fifth edition of the World Health Organization (WHO) classification of CNS tumors, revised in 2021, has been notably influenced by the integration of molecular results derived from DNA and RNA-based molecular methodologies, along with DNA methylation profiling [13]. These techniques have proven to be essential in characterizing CNS neoplasms and revealing crucial driver events, including oncogenic gene fusions. With the application of genome-wide and non-targeted methodologies for fusion detection, novel fusion events are being discovered in routine clinical diagnostic settings.

Pediatric low-grade gliomas (pLGG) and glioneuronal tumors (GNT) comprise over 30% of pediatric CNS tumors [3]. Within this category, GNTs pose a considerable diagnostic challenge because they lack consistent distinguishing histological characteristics. Several histological subtypes are acknowledged, yet in clinical practice, their differentiation is often challenging [25, 26].

A substantial number of pLGG/GNTs are associated with oncogenic fusion events. The most commonly observed fusions in pLGG/GNTs involve *BRAF*, *FGFR1*, *MYB*, and *MYBL1*, which result in up-regulation of the RAS-mitogen-activated protein kinase (RAS/MAPK) and PI3K pathways [15, 19–21]. Receptor tyrosine kinase (RTK) fusions, such as those involving *MET*, *ALK*, *ROS1*, and *NTRK*, drive a group of infantile hemispheric gliomas, but are generally rare in pLGG/GNTs, accounting for less than 5% of cases [4, 9, 21].

Mesenchymal–epithelial transition factor (*MET*) encodes an RTK which activates MAPK, PI3K/AKT, SRC, and STAT pathways to promote cell proliferation, invasion, and angiogenesis [12, 17, 23]. *MET* fusions, activating mutations, exon 14 skipping, and amplifications, leading to MET overexpression have been identified in a variety of human cancers [14, 28]. In the context of CNS tumors, *MET* fusions, with different 5’ partner genes, have been predominantly observed in high-grade gliomas, with a notable prevalence in infantile high-grade gliomas in the pediatric setting. However, *MET* fusions have not been commonly associated with pLGG/GNTs, and only several cases exist that describe their presence in low-grade GNTs [4, 8, 24].

We present two novel cases of pediatric glioneuronal tumors with a *CLIP2*::*MET* fusion detected by whole transcriptome sequencing (RNAseq), along with their clinical, pathologic, and molecular findings. While the *CLIP2*::*MET* fusion has been previously reported in three instances, including an adult glioneuronal tumor [8], a case of spontaneous regression of a congenital high-grade glioma [18], and at least two cases of infantile hemispheric high-grade glioma [1, 6, 9], this fusion has not been described in pediatric GNTs to date.

## Case presentation

### Case 1

**Fig. 1:**
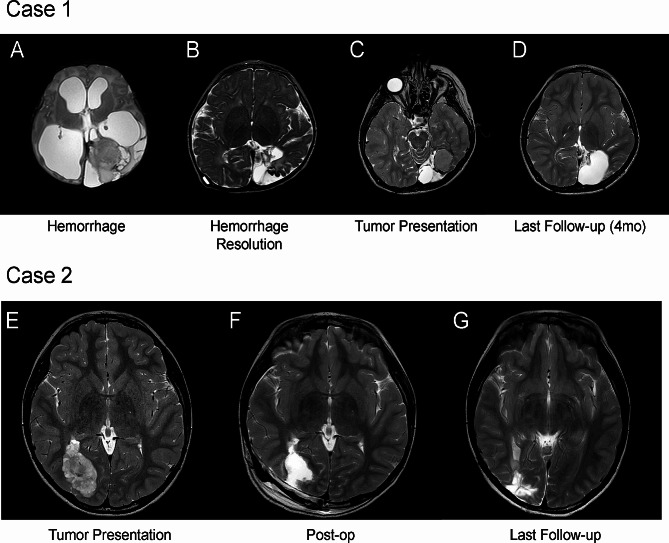
All images are T2 axial sequences with (**A**) displaying Case 1 with a left parieto-occipital intraparenchymal hematoma, IVH, and ventriculomegaly, (**B**) resolved intraparenchymal hematoma and post-shunt placement with residual hemosiderin staining, (**C**) spontaneous left parieto-occipital mass on pre-operative imaging found during surveillance MRI, and (**D**) displays the gross total resection at last follow-up (4 months). Figure 1E demonstrates Case 2 pre-operatively with a right parieto-occipital mass, (**F**) post-operative a near total resection was accomplished, and (**G**) a stable minimal residual mass at last follow-up (3-years) post-operatively

A 1-day-old full-term male with meconium aspiration syndrome presented with *Escherichia coli* sepsis, and initial cranial ultrasound demonstrated 4.8 × 5.3 cm intraparenchymal and intraventricular hemorrhage within the left occipital lobe (Fig. 1A). Once medically stabilized, an MRI/MRA was obtained and demonstrated increased ventriculomegaly with cerebrospinal fluid (CSF) septations and 4th ventricle outflow obstruction. At 3 weeks of age, the patient underwent a ventriculoperitoneal shunt (VPS) placement for hydrocephalus. At 16 months of age, his MRI was stable, and all hemorrhage was resolved without any sign of lesion or mass. (Fig. 1B). At the age of 29 months, he presented to the emergency department with emesis, and underwent revision of the shunt. Post-operative MRI confirmed stable ventricles, loss of parenchymal volume, and a rounded left occipital lesion at the site of the original hemorrhage. The patient was discharged and followed with imaging. At 4-years-old, surveillance imaging demonstrated slow to minimal growth of the lesion measuring 3.2 cm x 2.0 cm (AP x TV). Surgical treatment was pursued at that time at four years post-hemorrhage (Fig. 1C). He underwent gross total resection and did not receive adjuvant therapy. His last follow-up was at 6-months post-operative, where he remained disease-free and at neurological baseline (Fig. 1D).

## Pathologic findings

**Fig. 2:**
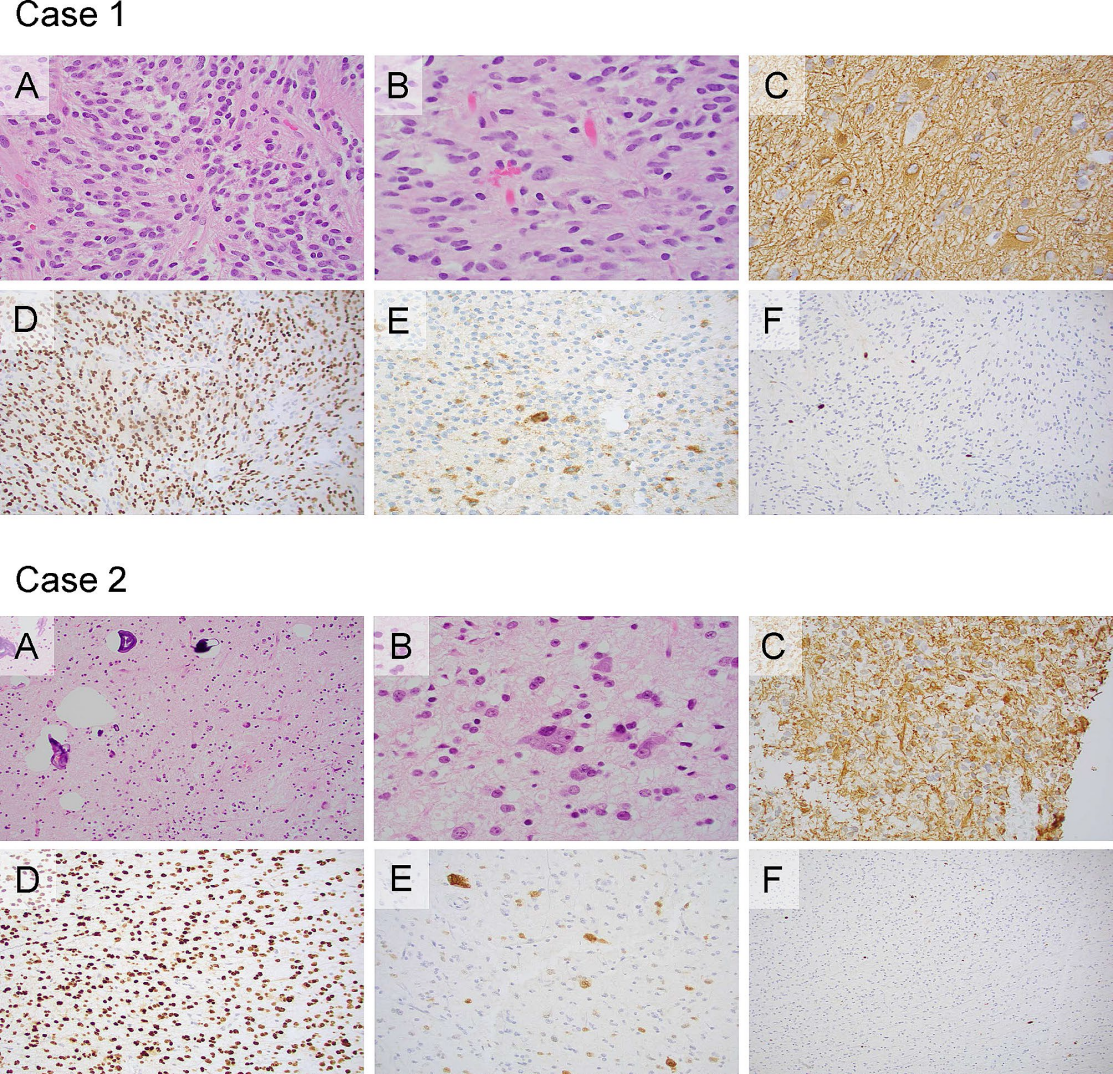
**Case 1 A** The tumor is comprised of a fairly bland population of neoplastic cells (H&E, 400X) **B**. Occasional multinucleated forms are present (H&E, 600 X). **C**. Many tumor cells express GFAP (GFAP, 600X) **D**. The vast majority of tumor cells are positive for OLIG2 (OLIG2, 200X). **E**. Scattered tumor cells are also positive for chromogranin (chromogranin, 400X). **F**. The Ki-67 labeling index was low (Ki-67, 200X). **Case 2 A** The tumor shows a mixture of bland mononuclear cells, and multinucleated cells with frequent microcalcifications. (H&E, 200X). **B**. **A** population of ganglioid cells is also present (H&E, 600X). **C**. A proportion of the tumor cells are positive for GFAP (GFAP, 400X). **D**. The tumors cell nuclei are diffusely positive for OLIG2 (OLIG2, 200X). E. NeuN is expressed in numerous cells within the tumor indicating neuronal differentiation (NeuN, 200X). **F**. The Ki-67 labeling index is low (Ki-67, 100X)

Microscopic examination showed a moderately to densely cellular tumor comprised mainly of bland neoplastic cells with rare scattered larger tumor cells with occasional multinucleation. Mitotic figures were rare and the Ki-67 labeling index was low (1-2%). Scattered microcalcifications and eosinophilic granular bodies were present. No necrosis or microvascular proliferation was present. There was focal infiltration of the adjacent brain parenchyma. The tumor cells were positive for glial markers (GFAP, OLIG2) and markers of neuronal differentiation (synaptophysin, chromogranin) were focally expressed within the tumor. The histomorphologic and immunophenotypic features were most consistent with a low-grade glial neoplasm with a focal neuronal component (Fig. 2). The tumor did not show immunohistochemical evidence of IDH1 R132H, ATRX, BRAF V600E, INI1, BCOR, or H3K27me3 alterations.

### Molecular findings

**Fig. 3:**
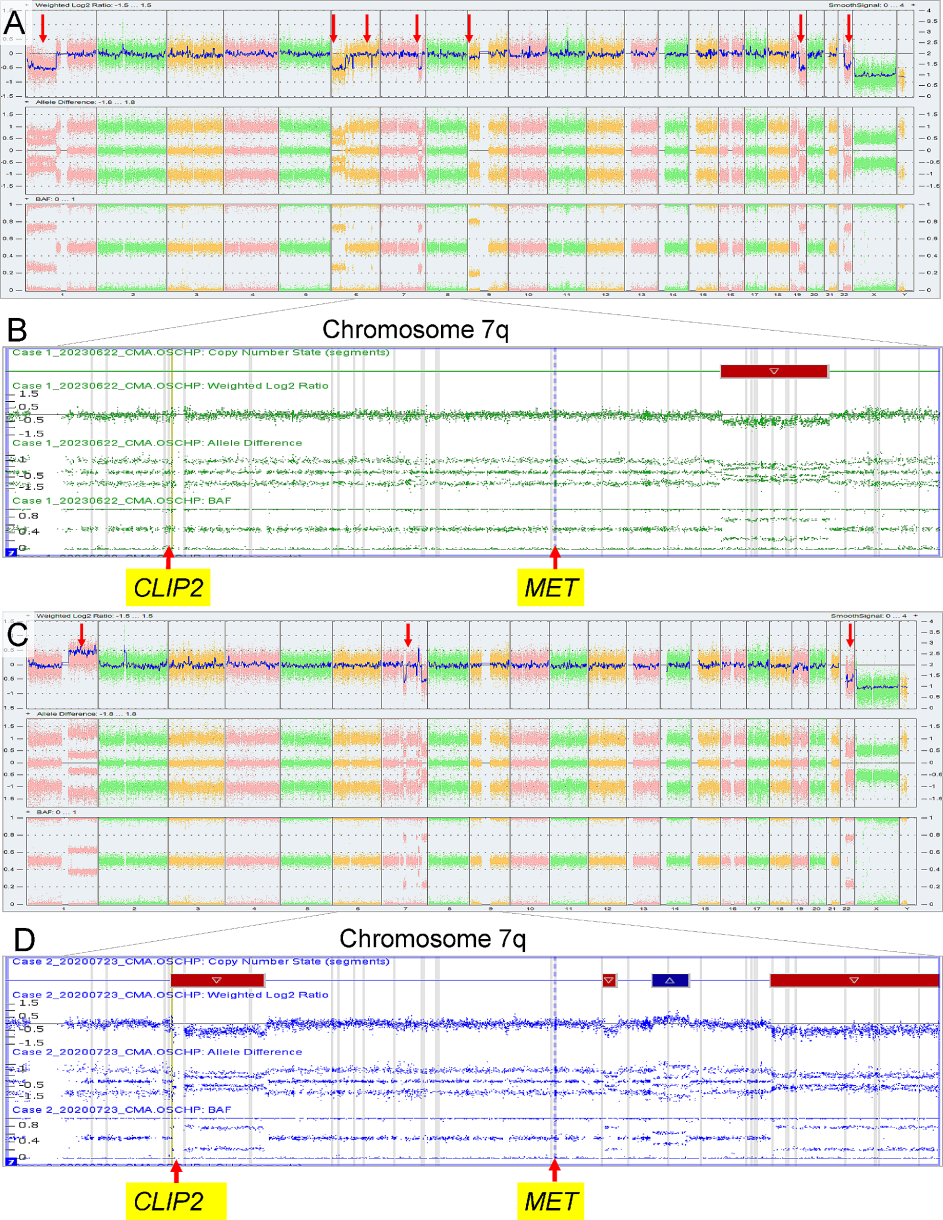
Chromosomal microarray analysis results on the tumor samples. (**A**) Whole-genome view from Case 1, from left to right, displaying chromosomes 1–22, followed by X and Y. Red arrows indicate copy number losses involving 1p, 6p, 9p, 19q, and 22q. (**B**) Chromosome 7q exhibited an interstitial deletion in the long arm. The breakpoints do not involve the *CLIP2* or *MET* genes, suggesting that the *CLIP2*::*MET* fusion likely results from rearrangements in a primarily balanced form. Note that the breakpoints in 1p and 19q were more distal than the typical 1p/19q co-deletions observed in oligodendrogliomas. (**C**) Whole-genome view from Case 2. Red arrows indicate gain of 1q, several copy number alterations in chromosome 7q, and a loss of chromosome 22. (**D**) Chromosome 7q showed several copy number alterations, including two interstitial deletions, an interstitial gain, and a terminal loss. Notably, the proximal breakpoint of the 7q11.23q21.11 deletion is within the *CLIP2* gene, indicating a potential rearrangement. The *MET* gene is not included in the copy number alterations observed in 7q.

**Fig. 4:**
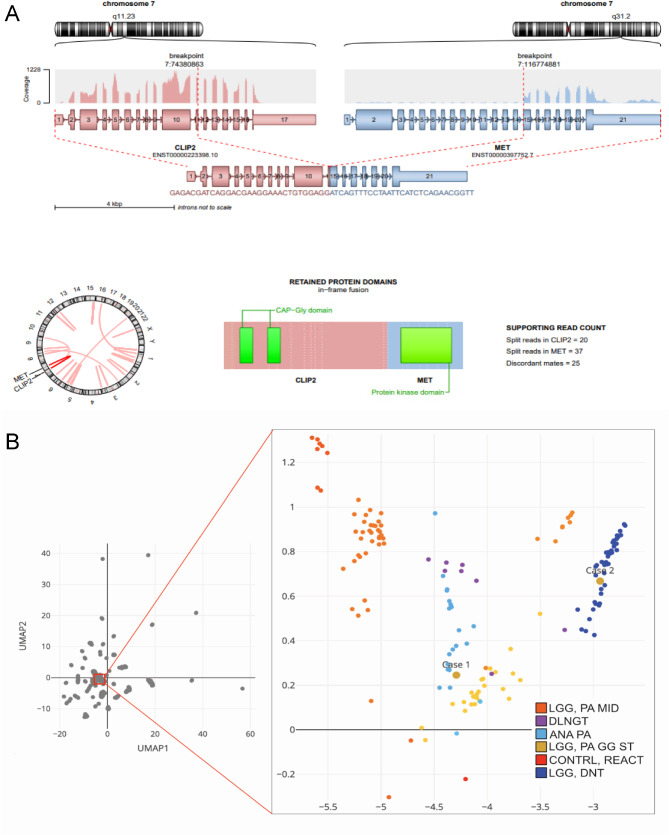
**A***CLIP2*::*MET* fusion identified in tumor samples by RNA-seq. Both tumors showed the *CLIP2*::*MET* fusion with the same exon-exon fusion junctions. The fusion occurs in-frame, resulting in the expression of a fusion protein encoded by the 5’ portion of the *CLIP2* gene (exons 1–11 out of a total of 17 exons) and the 3’ portion of the *MET* gene (exons 15–21 out of a total of 21 exons), which contains the protein kinase domain of *MET*. **B** DNA methylation results for both cases. A t-SNE map includes Case 1, Case 2, reference samples comprising low-grade glioma, subclass midline pilocytic astrocytoma (LGG, PA MID), diffuse leptomeningeal glioneuronal tumor (DLGNT), anaplastic pilocytic astrocytoma (ANA PA), low-grade glioma, subclass hemispheric pilocytic astrocytoma and ganglioglioma (LGG, PA GG ST), low-grade glioma, methylation class control tissue, reactive tumor microenvironment (CONTR, REACT), and dysembryoplastic neuroepithelial tumor (LGG, DNT). Case 1 clusters with the LGG, PA GG ST reference samples, although the tumor received a suggestive score (0.83) for the methylation class DLGNT by the random forest algorithm. Case 2 clusters with the LGG, DNT reference samples, but did not match to any specific methylation class using the random forest algorithm.

Chromosomal microarray analysis (CMA) of this tumor sample, using the OncoScan platform (Thermo Fisher Scientific), demonstrated an abnormal copy number profile with copy number losses encompassing most of the short arm of chromosome 1 (1p), 9p, a significant portion of 19q, and a substantial segment of 22q (Fig. 3A). There was also an interstitial deletion in chromosome 7q (Fig. 3B). Additionally, several nonconsecutive segmental deletions along the short and long arms of chromosome 6 were observed, along with the loss of most of 6p (Fig. 3A). Of note, the breakpoints in 1p and 19q were more distal than the typical 1p/19q co-deletions observed in oligodendrogliomas. OncoKids, a comprehensive DNA- and RNA-based next-generation sequencing panel [10], was negative for clinically significant DNA sequence variants, RNA fusions, and gene amplification events. Subsequent whole transcriptome RNA sequencing (RNAseq) analysis [5] of the tumor sample revealed a *CLIP2*::*MET* fusion (Fig. 4A). The fusion occurred in-frame, resulting in the expression of a fusion protein encoded by the 5’ portion of the *CLIP2* gene (exons 1–11 out of a total of 17 exons) and the 3’ portion of the *MET* gene (exons 15–21 out of a total of 21 exons), which contained the protein kinase domain of MET. This fusion is predicted to result in the upregulation of the MAPK signaling pathway [8]. As the deletion breakpoints in 7q do not involve the *CLIP2* or *MET* genes by CMA (Fig. 2A), the *CLIP2*::*MET* fusion likely results from rearrangements in a primarily balanced form. Further RNAseq analysis of the expression of the *MET* gene demonstrated higher expression of *MET* exons 15–21, which contained the tyrosine kinase domain, than that of exons 1–14 (**Supplemental Fig. 1**). By DNA methylation profiling, no definitive classification can be provided for this tumor, and the calibrated family and/or class scores were below the established in-house methylation class threshold of 0.88. However, the tumor received a suggestive class score of 0.83 for the methylation class “diffuse leptomeningeal glioneuronal tumor (DLGNT)”, wherein loss of 1p, with or without 19q loss, is prevalent [2, 7, 22]. Evaluation of the tumor using version 12.5 of the DKFZ classifier again yielded no match, with equivocal scores for “low grade glial/glioneuronal/neuroepithelial tumor” (0.46) and “diffuse glioneuronal tumor” (0.45) at the family level. The NCI’s Bethesda v2 classifier suggested a superfamily of “low grade glial/glioneuronal tumors” (mean score 0.645), and suggested class of “pilocytic astrocytoma, hemispheric” (mean score 0.991). In this case, the CMA findings of loss of significant portions of chromosomes 1p and 19q could support the diagnosis of DLGNT. Notably, no evidence of IDH1/2 mutation was detected. By UMAP analysis, the tumor clustered with the LGG, PA/GG ST (low grade glioma, subclass hemispheric pilocytic astrocytoma and ganglioglioma) reference samples (Fig. 4B). Overall, in the context of a *CLIP2*::*MET* fusion detected by RNAseq, the molecular findings are most consistent with a *CLIP2*::*MET* fusion-positive glioneuronal tumor.

**Table 1:** Clinical, pathologic, and molecular features of reported CNS tumors with CLIP2::MET fusion

	Case 1	Case 2	Chowdhury et al. 2020	Riedmeier et al. 2021	Stucklin et al. 2021*	Stucklin et al. 2021*
Clinical data						
Age at the diagnosis	4 yrs	8 yrs	30 yrs	1 mo	<1 mo	7 mo
Sex	Male	Male	Female	Male	Female	Male
Tumor location	Left occipital lobe	Right parieto-occipital lobe	Left parietal lobe	Left frontal/temporal lobe	Hemispheric, NOS	Hemispheric, NOS
Diagnosis at presentation	GNT	GNT	GNT	HGG	IHG	IHG
Treatment	GTR	GTR	GTR	Biopsy	Biopsy + Chemo	PR + Chemo
Follow-up time	0.5 yr	3 yrs	7 yrs	5 mo	0.7 yr	0.5 yr
Outcome	Alive	Alive	Alive	Alive	Alive	Alive
Pathologic features (IHC)						
GFAP	Positive in subpopulation	Positive	Focal positivity	Focal positivity	n/a	n/a
OLIG2	Positive	Diffuse nuclear positivity	Focal positivity	n/a	n/a	n/a
Synaptophysin	Granular positivity	Positive	Diffuse strong positivity	Negative	n/a	n/a
NeuN	Negative in tumor cells	Positive in scattered tumor cells	Positive in tumor cells	n/a	n/a	n/a
ATRX	Retained nuclear expression	Retained nuclear expression	Retained nuclear expression	Retained nuclear expression	n/a	n/a
Ki-67	Low index 1.07%	Low index 3%	Low index 0.4%	20%	n/a	n/a
H3K27me3	Retained nuclear expression	Retained nuclear expression	n/a	n/a	n/a	n/a
Molecular features						
Copy number alterations	Copy number losses involving 1p, 6p, 6q, 7q, 9p, 19q, and 22q	Gain of 1q, copy number alterations in 7q, and loss of 22q	Loss of 1p, gain of 1q, and copy number alterations in 7q	n/a	n/a	n/a
Fusion exon pair by RNA-seq	CLIP2 exon 11::MET exon 15	CLIP2 exon 11::MET exon 15	CLIP2 exon 12::MET exon 15	CLIP2::MET, exon number n/a	CLIP2 exon 12::MET exon 15	CLIP2 exon 12::MET exon 15
MET expression by RNA-seq	Higher MET expression in exons 15-21, including the tyrosine kinase domain, than in exons 1-14	Higher MET expression in exons 15-21, including the tyrosine kinase domain, than in exons 1-14	n/a	n/a	n/a	n/a
Methylation profiling	No match by RF, clustered with LGG, PA/GG ST in UMAP	No match by RF, clustered with LGG, DNT in UMAP	Clustered with LGG, DNT t-SNE map	Infantile hemispheric glioma	Infantile hemispheric glioma	Infantile hemispheric glioma

Legend: *Limited molecular and pathology data available for the two patients described by Stucklin et al., yr: year, mo: month, GNT: glioneuronal tumor, HGG: high-grade glioma, IHG: infantile hemispheric glioma, GTR: gross total resection, PR: partial resection, n/a: not available, RF: random forest algorithm, LGG, PA/GG ST: low grade glioma, subclass hemispheric pilocytic astrocytoma and ganglioglioma, LGG, DNT: low grade glioma, dysembryoplastic neuroepithelial tumor

### Case 2

An 8-year-old male patient was followed for new onset seizures. Preceding ictal symptoms, he had blurry vision, nausea, and vertigo. Presenting to our clinic, his ictal with episodes involve left head tilting, upward gaze, and bilateral eye twitching. MRI revealed a right parieto-occipital mass (3.7 cm x 5.1 cm x 2.9 cm) (Fig. 1E). Patient was prescribed anticonvulsant due to focal seizures and discussed surgical options.

Two weeks after being seen in clinic he underwent near-total surgical resection. The post-operative MRI revealed a small residual nodule (3–4 mm) (Fig. 1F). Preliminary diagnosis of tissue demonstrated a glioneuronal tumor with no post-operative complications. The patient did not receive any adjuvant therapy. At two-week follow-up, patient’s vision improved, improved from neurological baseline, and discontinued the anticonvulsants. Now, he undergoes annual surveillance imaging. At last follow-up three years post-surgery, patient remains clinically stable, with stable residual disease (Fig. 1G).

## Pathologic findings

Microscopic examination showed a tumor that was variably cellular and extensively infiltrative into the surrounding brain parenchyma. The neoplastic cells had variable morphology including bland monomorphic cells, rare bizarre cells, and frequent multinucleated forms that were admixed with ganglioid cells. The tumor was diffusely positive for glial markers (GFAP and OLIG2) and there was widespread expression of synaptophysin. NeuN was positive in scattered tumor cells supporting the presence of a neuronal component. Mitotic figures were rare, the Ki-67 labeling index was low (3%), and there was no necrosis. Additionally, there were numerous microcalcifications and rare eosinophilic granular bodies. Overall, the features were that of a low-grade glioneuronal tumor (Fig. 2). The differential diagnosis based on morphology and immunophenotype included ganglioglioma and diffuse glioneuronal tumor with oligodendroglioma-like features (DGONC). This tumor did not show immunohistochemical evidence of IDH1 R132H, ATRX or BRAF V600E alterations or histone (H3K27M and H3G34R) modifications.

### Molecular findings

CMA of the tumor sample exhibited an abnormal copy number profile with gain of 1q, loss of chromosome 22 (Fig. 3C), as well as several copy number alterations in 7q, including two interstitial deletions, an interstitial gain, and a terminal loss. Notably, the proximal breakpoint of the deletion in 7q11.23q21.11 was within the *CLIP2* gene, indicating a potential rearrangement. The *MET* gene was not included in copy number alterations observed in 7q (Fig. 3D). There was no evidence for monosomy 14 or 1p/19q co-deletion. OncoKids showed no established clinically significant sequence variants, gene fusions, or amplification events. Subsequent RNAseq analysis revealed the same *CLIP2*::*MET* fusion as seen in Case 1, with exon 11 of *CLIP2* fused to exon 15 of *MET*. The fusion protein contained the protein kinase domain of *MET*. The same *MET* gene expression pattern was noted in this tumor, with higher expression of *MET* exons 15–21 than that of exons 1–14, using the RNAseq data (Fig. 4). DNA methylation studies did not match this tumor to a known or specific methylation class using the random forest algorithm with the local version of DKFZ 11b4 classifier, but it clustered with the LGG, DNT (low grade glioma, dysembryoplastic neuroepithelial tumor) reference samples by UMAP analysis (Fig. 4B). Additional evaluation with the DKFZ v12.5 also showed no match, with equivocal superfamily scores very similar to Case 1 (0.55 vs. 0.43). The NCI’s Bethesda v2 classifier suggested a superfamily of “low grade glial/glioneuronal tumors” (mean score 0.813) and a suggested class of “glioneuronal tumor, KinF A” (mean score 0.869). The clinical, pathological, and molecular findings of both cases, along with those from published cases with the *CLIP2*::*MET* fusion, are summarized in Table 1.

## Discussion and conclusions

In CNS tumors, *MET* fusions with different 5’ partner genes, have been predominantly observed in high-grade gliomas and have been reported to demonstrate aggressive biological behavior [16, 29, 30]. In the pediatric setting, there is a notable prevalence of *MET* fusions in infantile high-grade gliomas [9, 11, 27]. However, *MET* fusions have not been commonly associated with low-grade gliomas or, even more rarely, glioneuronal tumors. In a study of 1,000 low-grade gliomas by Ryall et al., RTK fusions were identified in < 5% of the cases evaluated, and *MET* fusions in less than 1% of the cases [21]. To date, only several cases of *MET* fusions have been reported in low-grade GNTs [4, 8, 24].

Histologically, both of our cases demonstrated low-grade features, including the absence of mitotic activity and no evidence of microvascular proliferation. There was evidence of glial and neuronal differentiation based on morphology and immunohistochemical stains. High-grade features were not observed in our cases, whereas CLIP2::MET fusion cases previously described in the literature showed high cellularity, brisk mitotic activity, and microvascular proliferation [9, 18]. Only a single glioneuronal tumor reported by Chowdhury et al. showed similar low-grade features to our cases, characterized by a low Ki-67 labeling index [8].

The *CLIP2*::*MET* fusion was identified by RNAseq in both of our cases. Neither was initially detected by OncoKids, as expected, since RNA sequencing with OncoKids is a targeted approach, and novel fusions may be missed by these targeted methods. Incorporating a genome-wide analysis approach into routine clinical diagnostics is imperative for the identification of such fusions. While the exact breakpoints at the DNA level are unknown, the exon-exon pair of the fusion is identical between the two cases, with exon 11 of *CLIP2* fused with exon 15 of the *MET* gene. The resulting fusion is in-frame, contains the protein kinase domain of MET, and is predicted to result in the upregulation of the MAPK pathway for tumorigenesis [8]. By RNAseq gene expression analysis, the presence of the differentially expressed 3’ *MET* (exons 15–21) between 5’ *MET* (exons 1–14) further supported the oncogenic impact of this fusion.

Notably, methylation profiling of both tumors was consistently clustered with low-grade glial/glioneuronal tumors across classifiers, such that Case 1 was clustered with PA/GG and Case 2 clustered with DNT, although the specific subclass was indeterminate on multiple classifiers. Our cases, in conjunction with the previously reported cases with *CLIP2*::*MET* fusion, including IHGs, suggest a variable methylation pattern. Additional cases may be helpful in determining whether these *CLIP2*::*MET* fusion-positive tumors have a specific methylation pattern in different age groups, such as IHG in infants, LGG/GNT in pediatric patients, and variable histology in adults. Interestingly, the same methylation clustering pattern seen in Case 2 has also been previously identified in a DNT by Chowdhury et al., but in a 30-year-old female patient [8].

Both the *CLIP2* and *MET* genes are located in chromosome 7, with *CLIP2* being more proximal (7q11.23) and *MET* more distal (7q31.2). The fusion is thus considered to result from intrachromosomal rearrangement(s). It is worth noting that Case 2 had an interstitial deletion on chromosome 7, with a breakpoint located within the *CLIP2* gene, suggesting potential involvement of *CLIP2* by CMA analysis. However, the absence of copy number variants involving *CLIP2* or *MET* in chromosome 7 does not rule out the presence of the *CLIP2*::*MET* fusion, as seen in Case 1. Nonetheless, both cases had chromosome 7 copy number alterations, which may be secondary to the intrachromosomal rearrangement(s). Although information regarding copy number alterations in *CLIP2*::*MET* fusion-positive tumors is limited, due to the limited total number of cases, chromosomes 1, 7, and 22 copy number alterations appear to be common, as observed in both our cases and the GNT case reported by Chowdhury et al. [8].

The clinical course in both of our cases appears to follow that of low-grade tumors. In Case 1, the tumor presented post-hemorrhage, and the hydrocephalus was treated with a ventricular-peritoneal shunt. The patient then underwent one gross-total resection. In Case 2, the patient had been followed by neurology for his chronic eye twitching, and once symptoms became worse, a mass was visualized on MRI, and a single near-gross total resection was accomplished. Both patients improved from baseline clinical status at the last follow-up, and they are being followed with serial imaging. Neither is undergoing adjuvant therapy.

Stucklin et al. reported two infantile high-grade hemispheric gliomas with a *CLIP2*::*MET* fusion. The first case underwent two resections and chemotherapy, while the second case underwent one resection and chemotherapy. These cases were evaluated retrospectively, limiting the clinical data gathered and investigated. At present, Chowdhury et al. is the only case in the literature with histologic similarity to our cases; however, the tumor arose in a 30-year-old female with dysphasia and right arm pain, whereas our cases arose in pediatric patients with varied symptomatology. The group’s MRI findings did show similarities to our cases, with solid and cystic compartments noted in the left parietal lobe, although they do not describe any hemorrhage in their case of adult GNT with *CLIP2::MET* fusion. The patient underwent gross-total resection, and no adjuvant treatment was administered. The patient has remained in remission for 7 years.

Interestingly, Riedmeier et al. present clinical parallels to our Case 1 in their case report on infantile high-grade glioma with a *CLIP2*::*MET* fusion. The patient presented with hydrocephalic complications and IVH. The mass on MRI presented with high-grade features, and a biopsy was completed, which demonstrated a congenital anaplastic astrocytoma and glioblastoma. Without further intervention, the mass underwent spontaneous regression by a 10-week follow-up.

Prior reports of *CLIP2*::*MET* fusion have displayed tumors in various locations, including the frontal, temporal, occipital, and parietal lobes. These investigations on *CLIP2*::*MET* fusions have discernible differences in both histological and clinical attributes. Chowdhury et al. and our study are the only published reports for GNT with *CLIP2*::*MET* fusion, and all occurred, specifically in the occipital and parietal-occipital lobes, and presenting with seizure, ocular, and/or motor dysfunction. Both of our cases show a favorable outcome with surgical resection and without adjuvant therapy; however, more cases are needed to more firmly establish clinical outcomes of patients with GNT harboring this fusion.

### Electronic supplementary material

Below is the link to the electronic supplementary material.

**Supplemental Fig. 1** *MET* gene expression for both cases: X axis: each orange and blue dot from left to right represents *MET* exons 1–21; Y axis, log2 TPM FC (Transcripts Per Million Fold Change), log2 value of the TPM fold change, which represents the *MET* exon expression. *MET* exons 15–21, which contain the tyrosine kinase domain, showed higher expression than that of exons 1–14 for both tumors

## Data Availability

Not applicable.

## Abbreviations

ALK: Anaplastic lymphoma kinase
AP: Anterior-posterior
ATRX: Alpha-thalassemia/mental retardation, X-linked
CMA: Chromosomal microarray analysis
CNS: Central nervous system
CSF: Cerebrospinal fluid
DNT: Dysembryoplastic neuroepithelial tumour
FGFR1: Fibroblast growth factor receptor 1
GG: Ganglioglioma
GNT: Glioneuronal tumor
H3K27me3: Trimethylation of histone H3 at lysine 27
INI1: Integrase interactor 1
IPH: Intraparenchymal hemorrhage
IVH: Intraventricular hemorrhage
MET: Mesenchymal–epithelial transition factor
MRI: Magnetic resonance imaging
MYB/L1: Myeloblastosis proto-oncogene/like 1
NTRK: Neurotrophic tyrosine receptor kinase
PA: Pilocytic astrocytoma
PI3K: Phosphoinositide 3-kinases
P53: Tumor protein 53
pLGG: Pediatric low-grade glioma
RAS/MAPK: Ras/mitogen-activated protein kinase
RNAseq: RNA sequencing
RTK: Receptor tyrosine kinases
STAT: Signal transducers and activators of transcription
TV: Transverse
VPS: Ventriculoperitoneal shunt
