# Supplementary figures and images for: Clinical, pathologic, and genomic characteristics of two pediatric glioneuronal tumors with a CLIP2::MET fusion
Source: Acta Neuropathol Commun. 2024 Apr 22;12:63. doi: 10.1186/s40478-024-01776-1 (PMC11036580; doi:10.1186/s40478-024-01776-1)

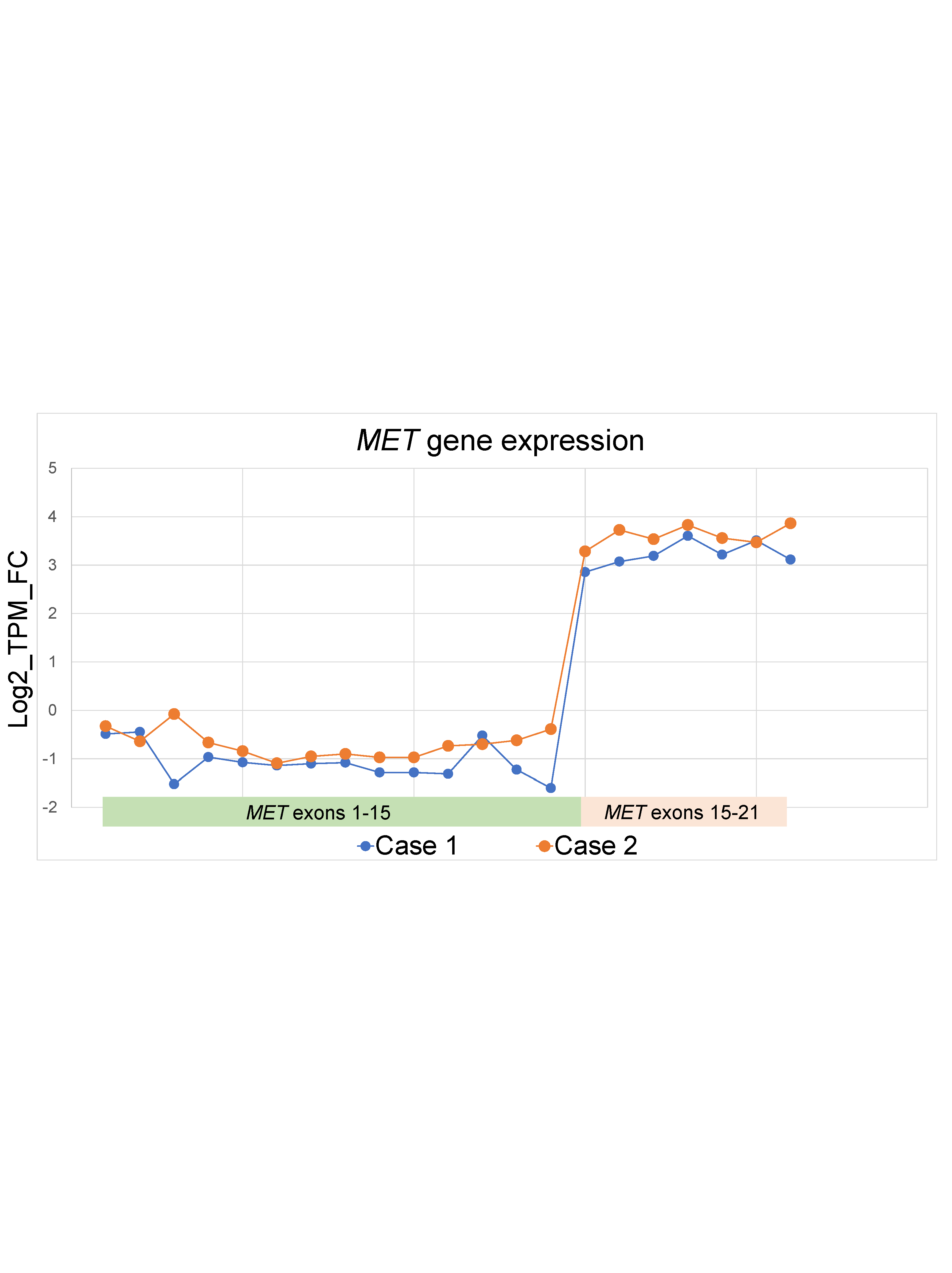

Supplement: Supplementary file 1 — Supplemental Fig. 1 MET gene expression for both cases: X axis: each orange and blue dot from left to right represents MET exons 1–21; Y axis, log2 TPM FC (Transcripts Per Million Fold Change), log2 value of the TPM fold change, which represents the MET exon expression. MET exons 15–21, which contain the tyrosine kinase domain, showed higher expression than that of exons 1–14 for both tumors [file 40478_2024_1776_MOESM1_ESM.tiff]
